# A Case of Methyl Ethyl Ketone Peroxide Ingestion Complicated by Rhabdomyolysis

**DOI:** 10.7759/cureus.12119

**Published:** 2020-12-17

**Authors:** Abhishek Dhir, Baldeep Kaur, Arshi Syal, Monica Gupta, Zainab Mehdi

**Affiliations:** 1 General Medicine, Government Medical College and Hospital, Chandigarh, IND; 2 Medicine, Government Medical College and Hospital, Chandigarh, IND

**Keywords:** mekp, poisoning, rhabdomyolysis, acute kidney injury, hemodialysis

## Abstract

Methyl ethyl ketone peroxide (MEKP) is a colorless-to-faintly-yellow liquid that is used as a cross-linking to harden plastics or resins in various industries. It is also an ingredient of paints, varnishes, and paint removers. Because of the high reactivity of MEKP, it is available only as a 40% to 60% solution in dimethyl phthalate or other phthalates. Post-ingestion, the spectrum of complications is vast, ranging from corrosive injury in the oral cavity and gastrointestinal tract to fulminant hepatic toxicity, sepsis, multi-organ failure, and disseminated intravascular coagulation. We report the case of a 40-year-old, self-employed, male worker in a lamination workshop, who presented with accidental ingestion of MEKP from an unlabeled container. He subsequently developed a multitude of complications, most noteworthy being rhabdomyolysis and in turn acute kidney injury. The patient was managed in the intensive care unit with supportive management and hemodialysis sessions; however, the patient succumbed to his illness, despite aggressive measures.

## Introduction

Methyl ethyl ketone peroxide (MEKP) is a potent oxidizing agent and corrosive used in many industries, principally as a plastic hardener and a catalyst. Exposure to this hazardous toxin can occur both accidentally or intentionally. Accidental ingestion is not uncommon, as the chemical is colorless with minimal odor and can be mistaken for water when kept in unlabeled containers. Although ingestion is rare, with approximately 29 cases reported to date [1], it is associated with a high degree of morbidity and mortality. Survivors often suffer from long term gastrointestinal sequelae due to fibrosis and stricture formation, although case reports also exist where they had excellent outcomes. Liver failure due to oxidative hepatocyte injury is the most significant single cause of mortality in literature. Reversible acute kidney injury (AKI) has been reported in very few case studies, mainly attributed to rhabdomyolysis [2]. Here, we present the case of a middle-aged male with accidental MEKP ingestion that ultimately proved fatal despite heroic measures. The presence of AKI secondary to rhabdomyolysis, which was unresponsive to aggressive supportive treatment and repeated hemodialysis, as well as the absence of hepatocyte injury (which is by far the most commonly reported adverse effect [2,3]), further adds to the uniqueness of this rare case.

## Case presentation

A 40-year-old, self-employed, male worker in a lamination workshop came to the hospital with an alleged history of accidental ingestion of approximately 100 ml of methyl ethyl ketone peroxide (MEKP) from an unlabeled container. He presented with complaints of swelling of the face and neck and multiple episodes of vomiting associated with traces of blood. On presentation, the patient had no complaints of shortness of breath, dizziness, muscle aches, or a decrease in urine output. On initial evaluation, the patient was found to be conscious and oriented to time, place, and person. Glasgow coma scale was 15/15. His pulse rate was 82 beats per minute, blood pressure was 110/80 mm of Hg, respiratory rate was 14 per minute and he was afebrile. Systemic examination, including respiratory, gastrointestinal, and cardiovascular was unremarkable. On local examination of the oral cavity, the oral mucosa was found to be edematous and had multiple ulcers extending up to the posterior pharyngeal wall. The tongue was ulcerated and coated. Diffuse neck swelling was present whereas laryngeal crepitus was absent and contour was normal. Initial management upon presentation included insertion of two wide-bore intravenous cannulas, urinary catheter, and infusion of intravenous fluids. A nasogastric tube was not inserted because of suspected upper gastrointestinal injury, marked by the presence of hematemesis. Intravenous infusions of N- N-acetylcysteine (NAC) and pantoprazole were commenced and continued for 48 hours. Intravenous ceftriaxone and metronidazole were also initiated. The laboratory findings made during the patient's hospitalization are listed in Table 1. Mild transaminitis was noted initially, which subsequently normalized by the second day, in striking contrast to renal function parameters, which deteriorated significantly over the course of his illness. Radiological investigations performed initially, including chest X-ray and abdominal ultrasound were unremarkable. Over the next two days, there was a deterioration of the patient’s condition, heralded by breathlessness and tachypnea. The patient developed profound respiratory difficulty and was unable to maintain normal oxygen saturation. The patient was shifted to the intensive care unit in view of impending type 1 respiratory failure where he was intubated and managed with mechanical ventilation. 

**Table 1:** Results of laboratory investigations performed during the hospital stay AST: aspartate aminotransferase, ALT: alanine transaminase, LDH: lactate dehydrogenase, CPK-MM: creatine kinase (muscle), SpO2: peripheral oxygen saturation

	Day 1	Day 3	Day 6	Day 10	Day 12
Hemoglobin (g/dL)	15.5		11.2	7.4	
Platelets (lac/mm3)	1.24		1.56	1.26	0.54
Total leucocyte count (10^9^ cells/L)	15.17		9	8.6	
Total serum bilirubin (mg/dL)	4.6	0.9	0.6	-	0.1
Conjugated bilirubin (mg/dL)	0.7	-	-	-	-
Alkaline phosphatase (U/L)	111	58	83		78
AST (U/L)	207	60	43		56
ALT (U/L)	90	32	21		32
Total protein	6.6	6	5.9		6
Albumin (g/dl)	3.1	3.1	3.2		3.1
Serum sodium (mEq/L)	143	149	154	147	
Serum potassium (mEq/L)	4.5	4.2	4.6	5.2	
Chloride (mEq/L)	105	113	121	109	
Blood urea (mg/dL)	70	199	243	205	
Serum creatinine (mg/dL)	2.2	5.9	7.1	6.9	
Uric acid (mg/dL)	7.2	8.1	7.2	-	
Calcium (mg/dL)	9.5	9.4	10.6	10	
Phosphorus (mg/dL)	3.4	3.5	3.7	5.4	
LDH (U/L)		626			
CPK-MM (U/L)		354			
Urine myoglobin (microgram/mL)		115			
Arterial Blood Gas Analysis					
pH	7.28	7.15	7.24	7.38	7.21
HCO3^-^	14.8	14.8	19.2	17.3	16
pCO2	32	43.8	123	29.8	41.6
pO2	108	98.1	97.3	96.4	81.7
SpO2 (%)	96.1	95.6	94.3	96	95.3
Lactate	1.4	1.6	1.3	1.6	0.8

**Figure 1:**
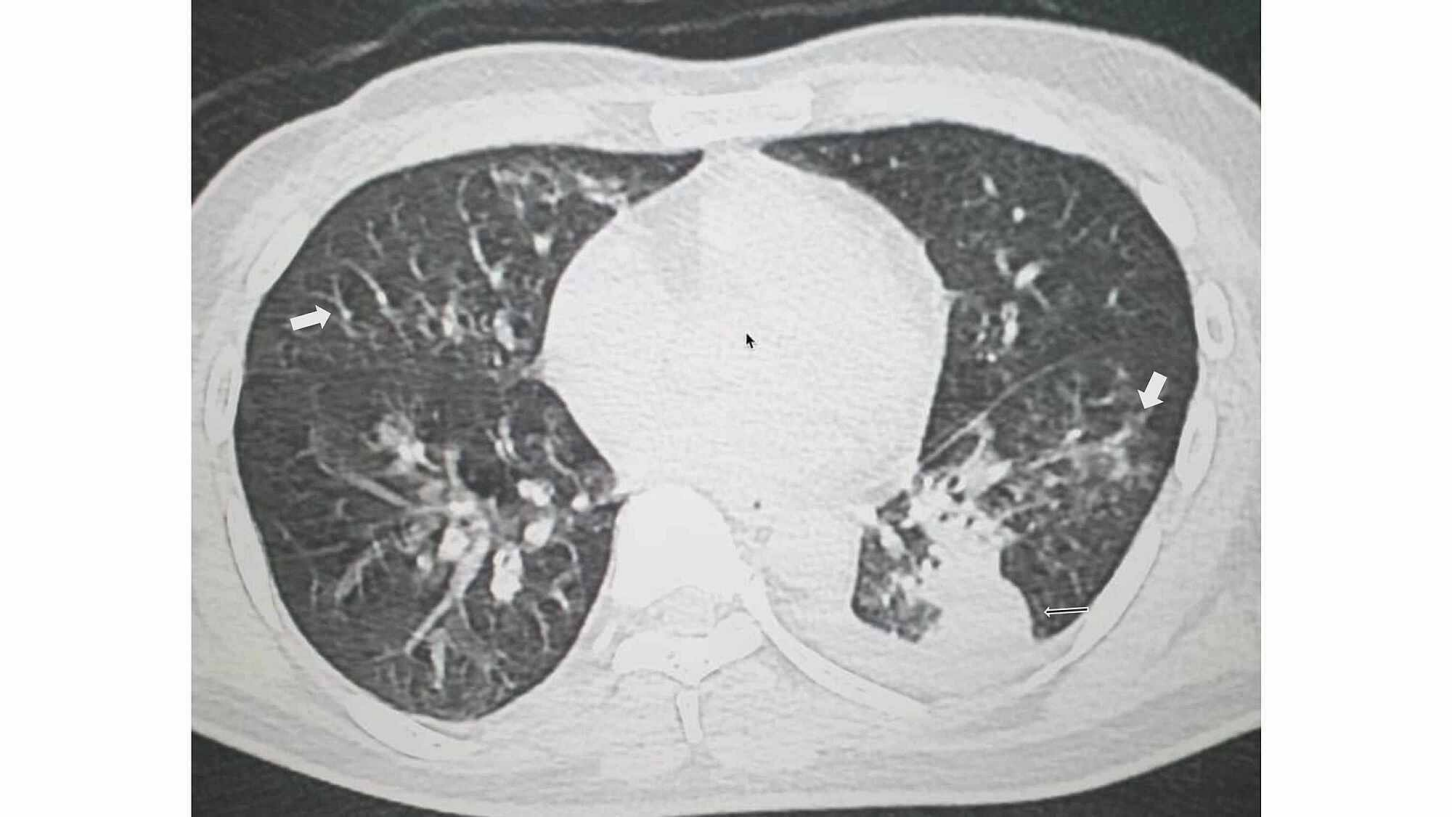
Non-contrast CT scan of the chest Bilateral ground-glass opacities with a tree-in-bud appearance are visible. A patch of consolidation in the left upper lobe and mild left pleural effusion can also be seen with the possibility of chemical or aspiration pneumonitis.

A non-contrast CT (NCCT) scan of the chest showed bilateral ground-glass opacities with a tree-in-bud appearance, suggesting the possibility of chemical pneumonitis (Figure 1). Both NCCT and ultrasound of the neck were unremarkable. Ophthalmological assessment of the fundus was unremarkable and no evidence of papillitis was seen. The subsequent fall in the urine output and the lab investigations depicting a worsening renal function profile raised the suspicion of rhabdomyolysis as the underlying culprit. The muscle enzymes (creatine phosphokinase, muscle, and lactate dehydrogenase) were found to be highly elevated and urine analysis was positive for myoglobin. The patient was subjected to hemodialysis sessions every alternate day in view of worsening renal function and high anion gap metabolic acidosis (Anion Gap 24.2). The patient’s condition progressively deteriorated despite aggressive treatment. He succumbed to his illness after a hospital stay of 12 days. 

## Discussion

The toxic oral dose of MEKP in dimethyl phthalate was estimated to be 50-100 ml [4]. This notorious chemical is known to produce free radicals and alkyl peroxide radicals which denature organic molecules, thereby resulting in peroxidation of unsaturated lipids, which are abundantly present in biological membranes [2]. This reaction is accelerated by the presence of iron in heme molecules in the biological systems [5, 6]. Case reports in literature portray an entire spectrum of clinical presentation. This largely depends upon the mode of the poisoning, the quantity of exposure, and the time taken to present to the hospital, to some extent [4]. Our patient presented to the hospital, after about eight hours of ingestion, thereby crucial time was lost.

MEKP on direct contact with the skin produces severe irritation and spillage into the eyes can lead to caustic damage. If ingested, it causes oral ulcers/ burns, although the extent of burns in the oral cavity is a poor predictor of disease [2]. Further, ingestion can cause pronounced ulceration of the gastrointestinal mucosa resulting in severe emesis. At the extreme of the spectrum, perforation and hemorrhage can arise, in turn leading to severe chemical peritonitis and sepsis. Endoscopic evaluation is recommended within the first 24 hours of ingestion and may be beneficial up to 96 hours, after which it should be avoided until after two weeks due to the risk of perforation [2]. In the subacute phase, poisoning can result in proximal intestinal obstruction due to mucosal edema, which usually sets in 24-48 hours after the insult, but tends to resolve on its own [2]. Over the long term, the scarring process may induce stenotic lesions in the gastrointestinal tract, which often requires therapy varying from esophageal dilatation to partial resections of the stomach and/or the proximal small bowel.

If inhaled, severe chemical pneumonitis tends to occur. MEKP can be aspirated resulting in proximal airway edema and obstruction, pneumonitis, and acute respiratory distress syndrome [7]. Our patient too had acute respiratory failure attributable to radiologically confirmed chemical pneumonitis.

Upon systemic absorption, the aforementioned free radicals in MEKP produce its deleterious effects on the hepatocytes, leading to extensive hepatic necrosis, potentially inducing acute liver failure, which has been reported as the primary culprit for the death of most patients alongside gastrointestinal complications, sepsis, and multi-organ failure. Van Enckevort et al. described the case fatality rate of this intoxication due to acute liver failure to be 26% [3]. Of the few cases reported in the literature, gastrointestinal and hepatic injury have been reported unanimously in almost all of them, which was in striking contrast to our case, as there was minimal evidence of hepatic injury. This may be attributed to early NAC administration, potentially responsible for preventing hepatocyte damage.

Reversible acute kidney injury (AKI) has been reported in very few studies of MEKP ingestion and is attributed mainly to rhabdomyolysis. In other studies, renal failure has also been attributed to hypotension secondary to massive dehydration as a direct result of MEKP toxicity [8]. Direct injury to the sarcolemma causing depletion of ATP within the muscle cells can result in increased intracellular calcium leading to uninhibited muscle contraction and ATP depletion, thereby creating a vicious cycle. The result is the destruction of the myocytes [9]. Myoglobin gets accumulated in the renal tubules and this is aggravated by volume depletion and renal vasoconstriction (a characteristic feature of rhabdomyolysis induced AKI). The major preventive and therapeutic approach for this disastrous sequence of events with rhabdomyolysis undoubtedly is early volume repletion, preferably isotonic saline [10]. Volume repletion with normal saline should be initiated at a rate of 400 ml per hour (200 to 1000 ml per hour according to the clinical scenario), with concurrent monitoring of central venous pressure (CVP) as was done in our case. Fluid resuscitation should be carried out to achieve the target urine output of approximately 3 ml per kilogram of body weight per hour (200 ml per hour). Hypocalcemia should be treated only if symptomatic (e.g., tetany or seizures) or in the case of severe hyperkalemia. Volume repletion should be continued until myoglobinuria is cleared (clear urine or dipstick negative for blood). Oliguria, as seen in our patient, is an indication of initiating renal replacement therapy [11].

Hemodialysis is highly beneficial in removing any accumulated organic acids, particularly formic acid, thus correcting metabolic acidosis and halting further neurologic and organ damage. An exact recommendation on the initiation and duration of dialysis is lacking. Van Enckevort et al. documented that it is reasonable to add dialysis to the therapeutic regime when and for as long as toxic metabolites can be demonstrated. If such metabolites cannot be measured, dialysis could be continued for a fixed period (e.g. every 48 hours) [3]. A recent study has demonstrated the benefit of continuous venovenous hemofiltration (CVVH) in effectively clearing myoglobin in patients with rhabdomyolysis and AKI and postulated that CVVH is potentially better than other modes of renal replacement therapy in this situation, although large trials are needed to validate this [10].

As with acute intoxication by any substance lacking a specific antidote, decontamination, management of the airway, breathing, and circulation along with supportive care remain the mainstay of treatment in MEKP poisoning. But the role of free radical scavengers, particularly NAC, vitamin E, and vitamin C in treating MEKP intoxication is noteworthy. A favorable outcome was documented when both oral and intravenous NAC was given in the first 24 hours. NAC is known to react with MEKP to convert it to inactive substances which can be easily excreted in urine [13]. The underlying mechanism involves the replenishment of glutathione stores and Vitamin E in the liver that combat free radicals [12]. Hence, early administration of NAC might prevent hepatocyte damage, as probably happened in our case. The recommended dose of NAC is presented in Table 2.

**Table 2:** The doses of N-acetylcysteine [11]

Dose	Duration of administration
Bolus I.V: 150 mg/kg N-acetylcysteine in a 5% glucose solution	15 min
Continuous I.V: 50 mg/kg N-acetylcysteine in a 5% glucose solution	8 hrs

## Conclusions

The ill-effects of MEKP ingestion can vary widely: from complete recovery without any long-term complications to death. Therefore, an integrated and aggressive approach for diagnosis and management is required to treat it. The most important steps include vigorous monitoring of vitals and laboratory parameters (chiefly liver, renal function profiles, urinalysis, and acid-base status), early aggressive fluid resuscitation, early administration of NAC which can potentially help scavenge the liver as in our case, and early anticipation of rhabdomyolysis by aggressive management with fluids and keeping a low threshold for hemodialysis. Supportive therapy in form of supplemental oxygen, proton pump inhibitors, and steroids can improve the outcomes. However, the treatment should be modified to suit the specifics of the case.
